# Musculoskeletal and Ergonomic Demands of the Pumping Maneuver in Laser-Class Sailing: An Integrated Biomechanical Analysis

**DOI:** 10.3390/sports14030113

**Published:** 2026-03-13

**Authors:** Carlotta Fontana, Nicola Laiola, Alessandro Naddeo, Rosaria Califano

**Affiliations:** Department of Industrial Engineering, University of Salerno, 84084 Salerno, Italy; cfontana@unisa.it (C.F.); anaddeo@unisa.it (A.N.)

**Keywords:** laser sailing, pumping maneuver, discomfort, musculoskeletal simulation, REBA ergonomic assessment, muscle activation

## Abstract

Background: Pumping in Laser-class sailing is a dynamic propulsion technique used in marginal wind conditions and characterized by repetitive, coordinated oscillations of the sailor–sail system. Despite its practical relevance, its biomechanical and ergonomic demands remain insufficiently characterized. Methods: A mixed-methods framework was applied combining questionnaire data, kinematic analysis, ergonomic assessment, and musculoskeletal modelling. Thirty-six competitive Laser sailors completed a Borg CR-10-based questionnaire on perceived discomfort/fatigue across body regions at predefined time points (during pumping, immediately after training, and the following day). A controlled land-based multi-angle video acquisition was used to reconstruct a standardized pumping posture and parameterize a digital human model in DELMIA^®^ for postural/kinematic analysis. Ergonomic risk was assessed using REBA, and muscle activity was estimated using the AnyBody^®^ Modeling System (simulation-derived normalized muscle activity across 129 muscles). Results: the simulation identified high neuromuscular demand in the trunk and shoulder complex, with several deep trunk stabilizers and the left latissimus dorsi reaching 100% modeled normalized muscle activity. Marked lateral asymmetry was observed, with right-sided trunk dominance and left-sided shoulder dominance. Kinematic analysis showed substantial joint excursions, with large lumbar motion amplitudes, while REBA yielded a score of 11 (Very-High Risk). Questionnaire data indicated a high prevalence of pumping-related musculoskeletal discomfort (72.2%), most frequently involving the lower back, shoulders, and knees. A dissociation was observed between modeled muscle activity and perceived fatigue, with the lower limbs rated as most fatigued despite lower modeled activation than the trunk. Conclusions: Findings identify the deep trunk stabilizers, latissimus dorsi, and lower extremities as key regions involved in pumping, with marked lateral asymmetry and high ergonomic risk. They support targeted training, injury-prevention, and ergonomic strategies to improve performance and reduce injury risk in competitive sailing.

## 1. Introduction

The Laser dinghy class has maintained its position as one of the most widely contested single-handed Olympic sailing disciplines for over five decades, attracting competitors across all levels of expertise and geographical regions [1]. The physical and technical demands placed upon Laser sailors are considerable, requiring not only refined boat-handling skills and tactical acumen but also exceptional muscular endurance, explosive power, and postural control. Within the repertoire of maneuvers executed during competitive racing, pumping occupies a distinctive position. This technique—characterized by rhythmic, coordinated oscillations of both sail and sailor’s body mass—serves to generate supplementary propulsion when natural wind conditions prove marginal or unstable. Whilst pumping is regulated under World Sailing Racing Rules of Sailing [2], permitting its use only under specific circumstances, mastery of this maneuver remains integral to competitive success, particularly in light and variable wind regimes.

From a biomechanical standpoint, pumping presents a complex motor task. The sailor must execute rapid, repetitive flexion–extension cycles involving the upper limbs, trunk, and lower limbs in a coordinated sequence, whilst simultaneously maintaining dynamic equilibrium on an inherently unstable platform [3]. These movements generate substantial musculoskeletal loads, with force transmission occurring through multiple kinetic chains and across numerous anatomical structures. The cyclical nature of pumping—alternating between concentric and eccentric muscle actions at high frequency—distinguishes it fundamentally from the more extensively studied hiking position. Hiking, by contrast, involves sustained isometric contraction of the knee and trunk extensors to counterbalance heeling moments, and has been the subject of considerable research attention over the past three decades [4,5]. Pumping, however, has received comparatively little systematic investigation, despite anecdotal reports from athletes and coaches suggesting that it imposes unique physiological stresses and may contribute to specific patterns of musculoskeletal discomfort and injury.

This gap in knowledge is particularly salient given emerging evidence from related aquatic sports suggesting that repetitive, high-intensity dynamic movements can precipitate early-onset muscular fatigue, metabolic disturbances including lactate accumulation, and micro-traumatic tissue damage [6]. Epidemiological data compiled by Neville and Folland indicate that sailing carries a non-negligible injury burden, with reported incidence rates of approximately 0.2 injuries per athlete per year among elite competitors [7]. Nathanson and colleagues documented injury and illness patterns through survey methodology, identifying musculoskeletal complaints as predominant concerns [8]. Specific anatomical regions appear particularly vulnerable: Kostański et al. reported elevated prevalence of lower back pain among youth Optimist sailors [9], whilst Jansen et al. investigated discomfort associated with prolonged hiking and proposed ergonomic interventions to enhance comfort and reduce injury risk [10].

The etiology of sailing-related musculoskeletal disorders likely reflects the interaction of multiple factors, including repetitive loading, sustained awkward postures, exposure to environmental stressors, and potentially inadequate physical conditioning or technical proficiency [11]. Pumping, with its distinctive biomechanical profile, may contribute to this injury burden through mechanisms that differ from those associated with hiking or other sailing maneuvers. The rapid acceleration and deceleration of body segments during pumping cycles generate substantial inertial forces, whilst the requirement for precise timing and coordination may expose sailors to elevated risk during phases of fatigue or suboptimal technique. Furthermore, the regulatory constraints governing pumping use—limiting its application to specific wind and wave conditions—mean that sailors may have fewer opportunities to develop technical mastery and physiological adaptation compared with more frequently practiced maneuvers.

Assessment of biomechanical loading and injury risk in occupational and sporting contexts has been advanced considerably through development of standardized ergonomic evaluation tools. The Rapid Entire Body Assessment (REBA) method, introduced by Hignett and McAtamney, provides a systematic framework for quantifying postural risk based on joint angles, force requirements, coupling characteristics, and activity duration [12]. REBA has been validated across diverse applications and demonstrates acceptable inter-rater reliability and sensitivity to intervention effects. Madani and Dababneh conducted a comprehensive literature review of REBA applications, confirming its utility for identifying high-risk postures and prioritizing ergonomic interventions [13]. Whilst REBA was originally developed for occupational settings, its principles are readily transferable to sports biomechanics, where similar concerns regarding repetitive loading and awkward postures arise.

Complementing subjective and observational assessment methods, computational biomechanical modelling has emerged as a powerful approach for estimating internal loading conditions that are difficult or impossible to measure directly. Digital human modelling (DHM) platforms enable reconstruction of complex movements from kinematic data, typically obtained through motion capture or video analysis, and subsequent calculation of joint reaction forces, muscle forces, and activation patterns through inverse dynamics and optimization algorithms. These computational tools offer particular advantages when studying dynamic, whole-body movements in ecologically valid environments where laboratory-based measurement would be impractical or would fundamentally alter the task being studied [14].

Despite the availability of these methodological approaches, no previous investigation has integrated subjective Discomfort assessment, kinematic analysis, ergonomic evaluation, and musculoskeletal simulation to provide a comprehensive characterization of pumping biomechanics in Laser sailing. This multidimensional gap constrains our understanding of the physiological and mechanical demands imposed by this maneuver, limits our capacity to identify specific risk factors for injury, and hinders development of evidence-informed training protocols and equipment design modifications.

The present study was designed to address these limitations through the application of a multifaceted analytical framework. The objectives were fourfold: (i) to quantify patterns of perceived muscular discomfort across different anatomical regions during and following the execution of the pumping maneuver; (ii) to characterize the kinematic and postural features of pumping through controlled land-based simulations and video-based analysis; (iii) to assess ergonomic risk associated with the maneuver using the Rapid Entire Body Assessment (REBA) method; and (iv) to estimate muscle activation patterns and joint loading through validated computational musculoskeletal modeling techniques. On the basis of the mechanical requirements of pumping, it was postulated that the upper limbs, trunk, and lumbar region would be subjected to the highest levels of discomfort and biomechanical loading, reflecting their central role in force generation, transmission, and postural stabilization during the maneuver [15,16]. The combined application of subjective assessment, kinematic analysis, ergonomic evaluation, and computational modeling was adopted to enable a comprehensive and methodologically robust characterization of the physical demands associated with the pumping maneuver.

## 2. Materials and Methods

### 2.1. Procedure

The methodological framework followed a sequential and integrated multi-platform approach designed. The study combined two-dimensional kinematic acquisition, three-dimensional digital human modeling (DHM), ergonomic risk assessment, and musculoskeletal simulation. The workflow began with controlled video acquisition of a standardized pumping posture under laboratory conditions, allowing extraction of joint kinematics. These kinematic data were subsequently used to parameterize anthropometrically scaled digital human models within DELMIA^®^ (version V5-6R2017, Dassault Systèmes, Vélizy-Villacoublay, France) for postural reconstruction and ergonomic evaluation. Finally, the reconstructed posture was exported to the AnyBody^®^ Modeling System™ (version 7.3, AnyBody Technology A/S, Aalborg, Denmark) to estimate internal muscle activation patterns using inverse dynamics.

**Figure 1:**
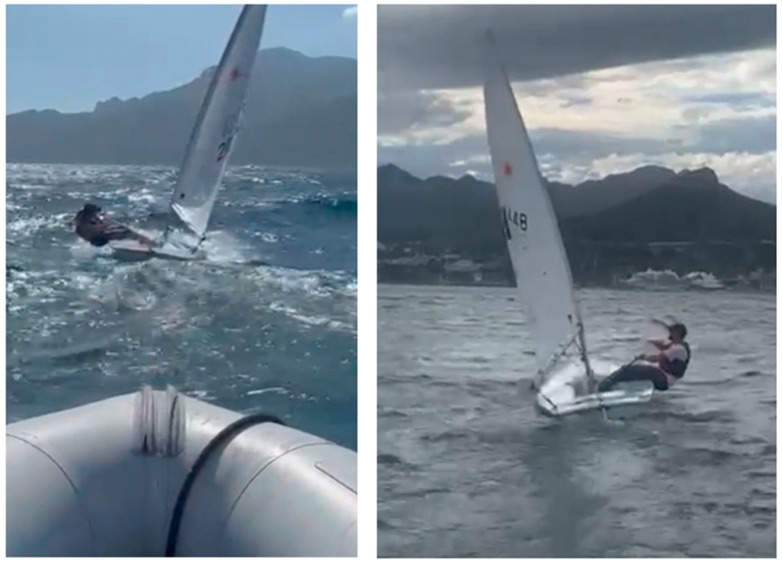
Representative on-water frames of the pumping maneuver in Laser-class sailing acquired during training sessions.

Athletes also performed training sessions and pumping maneuvers under real on-water sailing conditions. Subjective assessments (anthropometric questionnaire and perceived discomfort/fatigue ratings by body region) were collected in relation to the athletes’ training and recorded at predefined time points, including immediately after the on-water session (see Figure 1). In addition, supplementary on-water video recordings were analyzed frame by frame to qualitatively corroborate the postural patterns identified in the controlled land-based and simulation procedures.

### 2.2. Participants

A total of 36 Laser class sailors participated in the survey, comprising 28 males and 8 females. Only athletes with a minimum of five years of competitive sailing experience were included in the study. The sample size of the athletes was determined to ensure adequate representativeness of the target population, namely competitive Laser class sailors with a minimum of five years of experience at national and international levels. The athletes were selected based on their extensive racing experience and technical proficiency in the Laser class, ensuring a representative cohort of elite-level practitioners [17]. This criterion was defined as national-squad or equivalent professional-status sailors meeting at least two criteria of being among the top-20 national ranking or national podium, participation in Grade 1/World Championship regattas, and verified ≥5 years structured training with ≥200 on-water hours annually. The inclusion of high-performance sailors was essential to accurately assess the physical demands and discomfort patterns experienced during competitive sailing conditions. An offshore sub-sample of eight sailors participated, selected based on availability during scheduled sessions and predefined operational safety criteria (acceptable sea–weather limits, mandatory equipment, absence of recent injuries). This sub-sampling was motivated by logistical and safety constraints and does not alter the primary analyses conducted on the full sample.

### 2.3. Questionnaire and Data Analysis

Informed consent, in accordance with ethical standards of the University of Salerno, was obtained from all participants before their inclusion in the study. Each athlete received detailed information about the research protocol and voluntarily agreed to participate by signing the consent form. The experimental setup began with the request to participants (both competitive and pre-competitive sailors) to fill out an anonymized questionnaire. The questionnaire collected demographic information, including gender, age, and sailing experience, as well as anthropometric measurements such as height and hip height (see Table 1). Among anthropometric data collection, standing height and segmental heights were obtained on site, whereas body mass and foot size were self-reported in the questionnaire. No missing data were observed for any variable reported in Table 1.

Subjects were informed of the nature of the tests and their written consent, in accordance with the Ethical standards of the University of Salerno, was obtained. The assessment of perceived discomfort was conducted using the established Borg CR10 scale [18] which ranges from 1 to 10 and is widely recognized for quantifying subjective exertion and discomfort. For practical implementation in the field setting, a simplified five-point rating scale (from 1 = no discomfort to 5 = extreme discomfort) was employed, with values rescaled to maintain correspondence with the Borg scale indicators. This scaling factor allowed for standardized comparison across different conditions while preserving the clinical relevance of the Borg scale. Specifically, a rating of 3 on the simplified scale corresponded to approximately 5–6 (‘Strong’ to ‘Very Strong’) on the Borg CR10 scale, while a rating of 5 represented the maximum intensity (9–10 or ‘Extremely Strong’ on the Borg scale). To understand how discomfort evolved over time, the Discomfort assessment was performed under three distinct conditions: (A) during the hiking position, (B) immediately after sailing, and (C) the day following sailing [19].

### 2.4. Set Up

**Figure 2:**
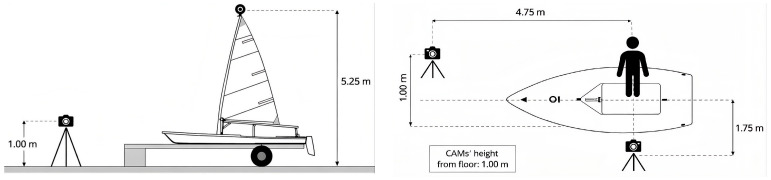
Lateral view—position of the cameras, on the (**left**); overhead view—position of the cameras, on the (**right**).

Due to geometric constraints during on-water filming, a standardized land-based acquisition protocol was implemented using a representative 50th percentile male sailor positioned on a stationary Laser vessel. The subject performed repeated pumping cycles while three synchronized high-definition cameras (60 fps) captured sagittal, frontal, and transverse views. Cameras were mounted on tripods, aligned perpendicular to the principal anatomical planes, and positioned at fixed height and distance to reduce parallax and perspective distortion (Figure 2).

Prior to recording, a simple geometric calibration was conducted using a rigid reference frame with known dimensions placed in the capture volume. In Kinovea^®^, two reference points were defined to scale pixel measurements to real distances. Horizontal alignment was verified with a digital inclinometer, and focal length remained constant across trials. Representative frames corresponding to the most demanding phase of pumping were selected, and anatomical landmarks were used to extract (in Kinovea^®^) neck, upper limbs, trunk, thighs, and legs angles for subsequent digital human model reconstruction.

### 2.5. Simulation of the Pumping Gesture

Due to the technical limitations in capturing high-quality, plane-orthogonal video footage during actual sailing—particularly in aligning cameras perpendicularly to the sagittal, transverse, and frontal planes of the athlete—the pumping gesture was reproduced through Delmia^®^ software (version V5-6R2017, Dassault Systèmes, Velizy-Villacoublay, France).

This computational platform facilitated the construction of a dimensionally accurate Laser class vessel (in accordance with ILCA 2024 specifications) and the digital human models (DHMs), anthropometrically scaled on participants’ body measurements.

This virtual ergonomics/DHM environment system has been applied and benchmarked for posture-based ergonomic assessment and task simulation in previous studies [20,21]. A REBA analysis was performed to evaluate the ergonomic risk associated with the most critical posture observed during the pumping gesture. The REBA method was chosen for its suitability in analyzing dynamic postures involving both upper and lower limbs, which is characteristic of the pumping action in sailing. The analysis was conducted using the posture identified as the most biomechanically demanding, based on joint angles obtained from the Delmia^®^ simulation. The resulting REBA score provides an estimate of the level of musculoskeletal risk associated with the gesture, supporting the interpretation of discomfort data collected in the survey phase and offering insights into potential injury risk or ergonomic improvements.

### 2.6. Muscle Activation Analysis

Muscle activation data were collected using AnyBody^®^, a validated musculoskeletal modeling software that enables detailed analysis of internal body mechanics [22]. A representative pumping posture was recreated in the software based on kinematic data collected from experienced competitive sailors. The forces applied during the simulation of the pumping maneuver were measured using a dynamometer and ranged from 183 to 307 N (approximately 19–31 kg); therefore, the mean value was used in the model simulation. The AnyBody^®^ inverse-dynamics model calculated muscle activation levels, expressed as a percentage of maximum voluntary contraction (%MVC), for 129 muscle actuators spanning the trunk, upper-limb, and lower-limb regions; for reporting, only the actuators with the highest simulated activity during pumping are reported.

## 3. Results

### 3.1. Subjective Evaluation

**Figure 3:**
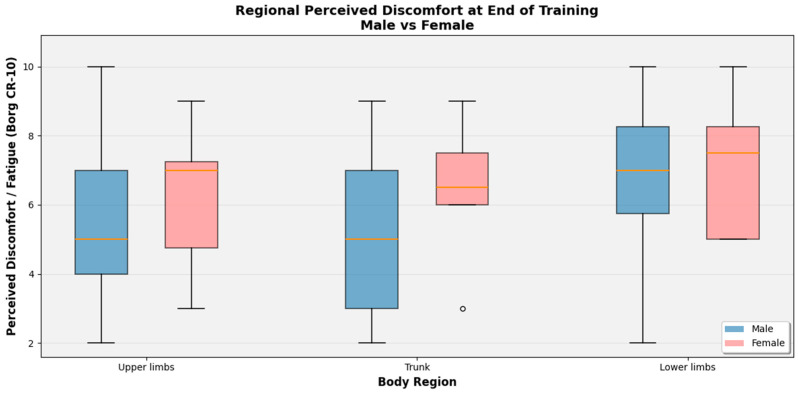
Regional perceived discomfort at the end of training in sailing athletes, stratified by gender.

Across all three body regions, females tended to report higher regional discomfort than males at the end of training, with a distribution more concentrated toward the upper range of the Borg CR-10 scale (Figure 3).

In the upper limbs, female medians clustered around 7, whereas males centered closer to 5, with a wider spread that included lower scores, suggesting a subset of male sailors experienced only moderate fatigue. A similar pattern was evident at the trunk: male ratings were more dispersed from low to high values, while female ratings were tightly grouped around 6–7, indicating consistently elevated trunk discomfort in women. In the lower limbs both genders reported the greatest fatigue, but again female scores were slightly higher and less variable, with most values between 7 and 9, whereas males showed a broader distribution extending down to lower levels of perceived effort. Taken together, these distributions suggest that, for a comparable training load, female athletes experienced more homogeneous and generally higher regional muscular fatigue, particularly in the upper body and trunk, while male athletes showed greater inter-individual variability with a subset reporting relatively lower discomfort.

**Figure 4:**
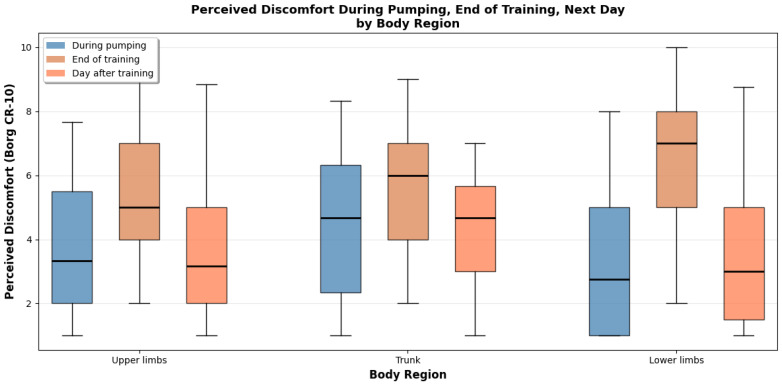
Perceived regional discomfort in sailing athletes during pumping, at the end of training, and on the day following training.

**Figure 5:**
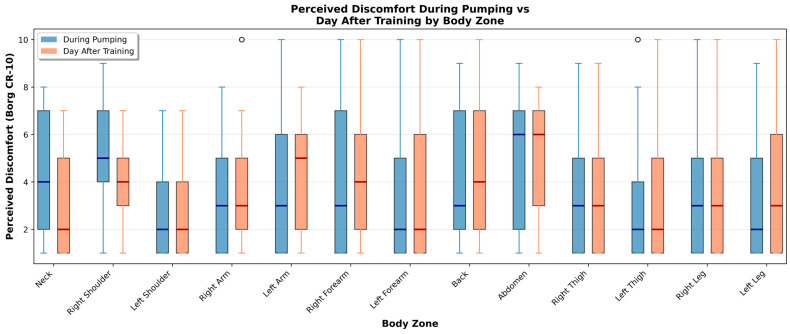
Perceived discomfort during pumping and on the day following training across specific body zones in sailing athletes.

Box plots illustrating the distribution of discomfort scores in each scenario are presented in Figure 4 and Figure 5.

During pumping, the body regions reporting the highest levels of discomfort were both quadriceps, hamstrings, abdominals, and both arms. This is consistent with the biomechanical demands of the pumping motion, which strongly engages both upper and lower body musculature. Conversely, regions such as the head, neck, and glutes were associated with lower discomfort levels.

Immediately after sailing, athletes reported increased overall discomfort, likely due to acute muscle fatigue.

The day after training, discomfort remained elevated in the quadriceps, hamstrings, abdominals, and arms, suggesting the persistence of delayed onset muscle soreness in the most heavily used muscle groups.

Detailed analysis of perceived muscular engagement across body zones during pumping maneuvers identified substantial regional variation. Table 2 summarizes perceived muscular engagement across body zones during pumping maneuvers (Borg CR-10), including ratings collected during pumping and on the day after training. During the execution of pumping, discomfort scores tended to be moderate and varied considerably across zones, with median values typically falling between 3 and 5 on the Borg CR-10 scale. In contrast, ratings collected the day after training were systematically higher and more homogeneous, indicating that delayed-onset muscular fatigue was more pronounced than the acute discomfort experienced during the activity. The right shoulder demonstrated the highest mean engagement score (5.36 ± 2.34), followed by the abdomen (4.81 ± 2.92), neck (4.30 ± 2.34), back (4.19 ± 2.72), and right forearm (3.86 ± 2.95). Conversely, the left leg (3.36 ± 2.93), left thigh (2.89 ± 2.45), and right leg (3.36 ± 2.53) exhibited the lowest engagement scores during pumping. This asymmetric pattern reflects the biomechanical asymmetry inherent to pumping technique, wherein the dominant upper limb and ipsilateral shoulder complex bear disproportionate mechanical load.

Comparison of perceived muscular engagement during pumping with residual fatigue reported on the subsequent day revealed notable temporal dynamics. Whilst certain zones, particularly the shoulders and back, demonstrated elevated engagement during pumping, the lower limbs exhibited disproportionately higher fatigue scores on the following day. Furthermore, the abdomen and trunk regions demonstrated relatively consistent scores across both time points, indicating sustained muscular demand throughout the training session and into the recovery period. A substantial majority of participants (91.7%) reported also that perceived exertion varied considerably as a function of meteorological conditions, particularly wind velocity. The primary factor attributed to increased exertion under high-wind conditions was the augmented force required to execute pumping maneuvers, followed by increased frequency of pumping actions.

### 3.2. Objective Evaluation

**Figure 6:**
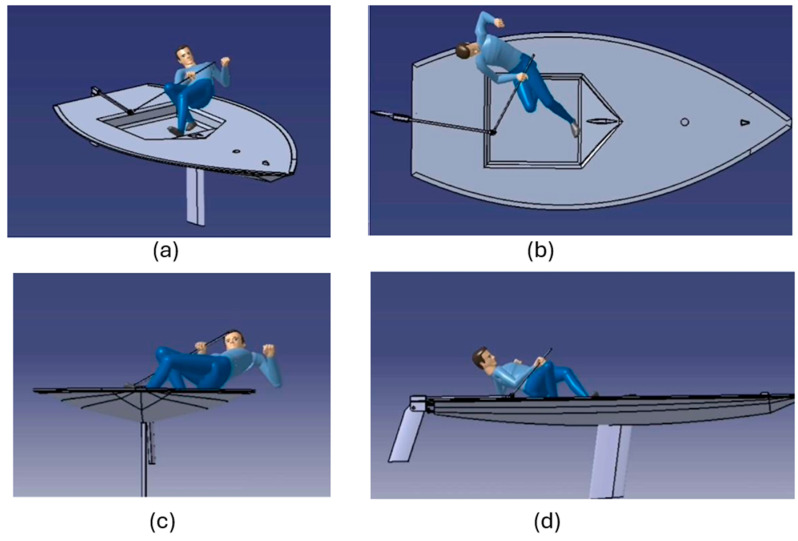
Representative frames extracted from the Delmia^®^ video simulation of the pumping maneuver: (**a**) oblique view, (**b**) top view, (**c**) front/oblique view, and (**d**) side view of the simulated sailor–boat system.

Four digital simulations of the pumping maneuver were conducted in Delmia^®^ using anthropometrically scaled digital human models representing a 50th percentile male (Figure 6a–d).

The simulations enabled quantitative evaluation of the principal joint angles associated with the pumping maneuver using the Delmia^®^ Posture Editor. Table 3 summarizes the minimum, mean, and maximum values of the main joint angles, as well as the range of motion (ROM), for the 50th percentile male model across a complete pumping cycle. These parameters provide an objective description of the postural demands imposed by the maneuver across the upper limbs, trunk, and lower limbs.

The analysis of joint kinematics across all anthropometric percentiles led to several observations. First, the magnitude of quadriceps flexion angles indicates a high level of mechanical demand on the knee extensor musculature, suggesting a potential source of discomfort during pumping. Similarly, the large variability observed in arm and forearm flexion angles reflects the dynamic nature of upper-limb involvement, in agreement with the elevated discomfort reported in the survey during active pumping. Second, trunk axial rotation exhibited a markedly negative minimum value with a high absolute magnitude, indicating that the trunk is frequently positioned in non-neutral, rotated postures that may contribute to discomfort and increased mechanical strain in the lumbar region. The remaining joint angles showed relatively limited variability and lower absolute values, consistent with the lower levels of perceived discomfort reported for these regions in the questionnaire data.

#### 3.2.1. Ergonomic Risk Assessment

To evaluate the ergonomic risk associated with the pumping maneuver, a Rapid Entire Body Assessment was performed [23].

For Group A (neck, trunk, and legs), the neck flexion angle ranged between 0° and 20°, corresponding to a score of 1, while the trunk inclination angle also fell within the 0–20° range, yielding a score of 2. The lower limbs were characterized by bilateral weight support (score 1) combined with knee flexion exceeding 60° (score 3). These values resulted in an initial Group A score of 4. Considering the rapid development of force, a Load/Force Score of 3 was assigned, resulting in a final Score A of 7.

For Group B (upper arms, lower arms, and wrists), the upper arm posture ranged between 0° and 45° of flexion, corresponding to a base score of 2, which was increased to 4 due to shoulder elevation and abduction. The lower arm flexion angle ranged between 60° and 100°, yielding a score of 1, while wrist angles remained below 15°, also corresponding to a score of 1. Combining these values after accounting for the grip score, Score B was 5. Scores A and B were combined and considering the repetitive and dynamic nature of the task, the final REBA score was 11, corresponding to a “very high risk” classification and indicating the need for immediate corrective and preventive interventions.

#### 3.2.2. Biomechanical Modeling

Analysis of the muscle activation data revealed distinct patterns of muscular recruitment during the pumping maneuver. Table 4 summarizes the activation percentages of the most activated muscles, grouped by body region. The muscle activation values reported from AnyBody^®^ refer to simulation-derived normalized muscle activity. Specifically, muscle activity was expressed as the ratio between predicted muscle force and the model-estimated current muscle strength. Accordingly, a value of 100% indicates that the predicted force reached the modeled force capacity for that muscle under the analyzed conditions and not the maximum exercisable force by the muscle itself.

As shown in Table 4, the analysis identified the trunk musculature as the region of highest activation, with several deep stabilizers reaching maximal normalized activation (100.0%). Specifically, the right-side multifidus (100.0%), bilateral psoas major (100.0% right, 100.0% left), right quadratus lumborum (100.0%), bilateral oblique muscles (100.0% external oblique right, 100.0% internal oblique left), and right semispinalis (100.0%) demonstrated peak engagement. The right erector spinae exhibited 91.8% activation, while the left erector spinae showed 58.7% activation, reflecting pronounced lateral asymmetry in spinal stabilization demands. The shoulder girdle musculature demonstrated substantial activation predominantly on the left side, with the left latissimus dorsi reaching maximal activation (100.0%). The left rotator cuff muscles, including deltoid (56.2%), infraspinatus (56.2%), and supraspinatus (56.2%), exhibited moderate-to-high activation levels. The left serratus anterior (43.2%) and trapezius (43.2%) showed moderate engagement consistent with scapular stabilization requirements. This left-sided shoulder activation pattern contrasts with the right-sided trunk dominance, reflecting the asymmetric postural and force transmission demands of the pumping technique.

Upper extremity musculature exhibited moderate activation, with the left biceps brachii (56.2%) and left triceps brachii (56.2%) demonstrating comparable engagement levels. The rectus abdominis (34.1%) and transversus abdominis (32.5%) showed moderate activation, contributing to anterior core stability. The left sternocleidomastoid (33.1%) exhibited activation consistent with cervical stabilization and head positioning demands.

Lower extremity musculature demonstrated moderate activation across multiple muscle groups, with the right tibialis anterior (45.5%), peroneus tertius (45.5%), semimembranosus (45.5%), biceps femoris (45.5%), sartorius (45.5%), tensor fasciae latae (45.5%), gracilis (45.5%), plantaris (45.5%), and popliteus (45.5%) all exhibiting similar activation levels. The right gastrocnemius (26.6%) and hip stabilizers including gluteus medius (26.6%), gluteus minimus (26.6%), iliacus (26.6%), and piriformis (26.6%) showed lower but sustained activation.

**Figure 7:**
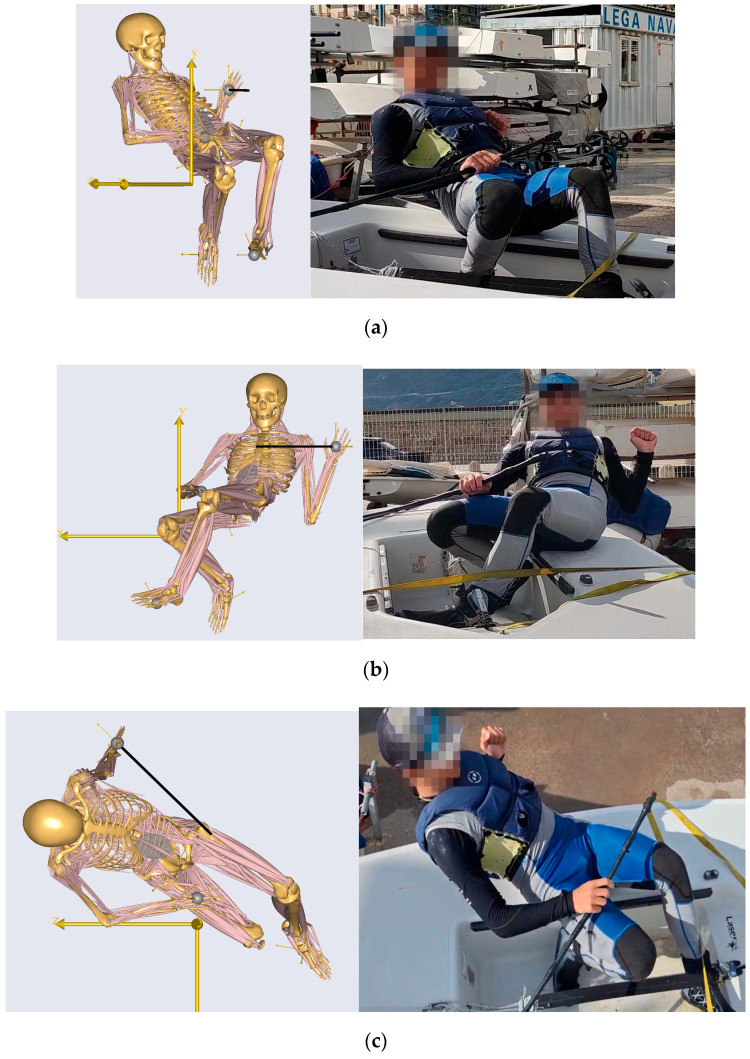
Qualitative visual validation of the digital human model during the pumping maneuver. Panels (**a**–**c**) show lateral, frontal, and top viewpoints, respectively, comparing the AnyBody^®^ simulated posture (**left**) with representative land-based posture reproduction frames acquired during the controlled protocol (**right**).

To visually validate the fidelity of the digital human model, a qualitative comparison was performed between the simulated pumping posture and representative land-based posture reproductions acquired during the controlled protocol. Figure 7a–c juxtaposes the AnyBody^®^ simulation output (left) with the corresponding experimental frames (right) from lateral, frontal, and top viewpoints. Across panels, the overall body configuration, trunk inclination, shoulder elevation, and lower-limb positioning observed in the AnyBody^®^ simulation closely resemble those recorded during on-water pumping.

The activation pattern revealed pronounced lateral asymmetry, with right-sided dominance in trunk musculature and left-sided dominance in shoulder girdle musculature. This asymmetric loading reflects the biomechanical constraints of the pumping technique, in which the sailor maintains an asymmetric stance with differential force transmission through the right and left sides of the body. The maximal activation observed in multiple deep trunk stabilizers indicates that these muscles operate at or near their physiological capacity during pumping, suggesting substantial neuromuscular demand and potential fatigue accumulation with repeated execution.

The high activation of deep spinal stabilizers in conjunction with the global trunk muscles indicates coordinated engagement of both local and global stabilization systems. This pattern is consistent with the biomechanical requirements of maintaining spinal stability while generating and transmitting forces through the kinetic chain during the dynamic pumping motion. The moderate lower extremity activation suggests that while these muscles contribute to postural stability and force generation, they do not approach the same relative intensity as the trunk and shoulder musculature.

## 4. Discussion

The biomechanical analysis of the pumping maneuver in Laser-class sailing provides an integrated view of the neuromuscular, kinematic, postural, and ergonomic demands associated with this technique.

The musculoskeletal simulation identified a high demand on the trunk stabilizing system, with several deep spinal and trunk muscles reaching very high model-derived normalized activity, together with substantial activation of the shoulder–latissimus complex. A marked lateral asymmetry was also observed, characterized by right-sided dominance in trunk musculature and left-sided dominance in the shoulder girdle. Rather than reflecting isolated muscle overload, this pattern suggests a coordinated, asymmetrical strategy for force generation and transmission during pumping, in which trunk stabilization and upper-body force transfer are tightly coupled.

From a functional perspective, the concurrent involvement of deep stabilizers and global trunk muscles indicates that pumping requires both segmental spinal control and whole-body force transmission along the kinetic chain. This combined demand supports the interpretation of pumping as a mechanically demanding maneuver, particularly for the trunk–shoulder complex.

When considered alongside the questionnaire data, the simulation results reveal an apparent dissociation between objectively estimated muscle activity and subjectively perceived fatigue. Sailors reported the greatest perceived fatigue in the lower limbs, followed by the upper limbs and trunk, whereas the simulation identified the trunk musculature as the most highly loaded region. This pattern should not be interpreted as a contradiction between objective and subjective findings, but rather as evidence that the two measures capture complementary dimensions of pumping-related load: the musculoskeletal model provides simulation-derived normalized muscle activity under a standardized task configuration, whereas the questionnaire reflects a time-integrated perceptual response that includes cumulative effort, discomfort, and recovery-related sensations. Accordingly, contemporary models of perceived exertion emphasize that fatigue perception emerges from the integration of central motor command and afferent feedback, and therefore may not scale linearly with local muscle activity estimated by simulation [24].

A plausible explanation for the greater perceived lower-limb fatigue, despite only moderate modeled activation, is the combined effect of prolonged postural stabilization, repeated isometric contractions, and cumulative metabolic demand during pumping and sailing more broadly. In practice, the lower limbs contribute continuously to balance control and body-position maintenance on an unstable platform, and this time-under-tension may generate a disproportionate perceptual burden relative to peak modeled activity. In addition, reactive stabilization demands associated with wave action, wind variability, and boat motion may further increase lower-limb fatigue during real sailing conditions, but are only partially represented in the standardized simulation framework.

By contrast, the trunk musculature, despite exhibiting the highest modeled activity, received comparatively lower subjective fatigue ratings. This may reflect the functional specialization of deep spinal stabilizers for tonic postural control, including greater fatigue resistance and lower perceptual salience of sustained activation. This interpretation is consistent with evidence that the lumbar multifidus is structurally and functionally specialized for tonic spinal stabilization, with properties compatible with sustained activation and relatively high fatigue resistance [25].

Moreover, proprioceptive and afferent feedback from deep trunk musculature may contribute differently to conscious fatigue perception than feedback from more superficial limb musculature. In particular, the lumbar multifidus has been reported to exhibit a prominent proprioceptive role (e.g., high muscle-spindle density), which may modulate sensory salience and the conscious attribution of fatigue during stabilisation-dominant tasks [26]. The relatively modest perceived trunk engagement may also indicate that experienced sailors develop efficient neuromuscular strategies that attenuate the sensation of effort even when operating near high mechanical demand. Finally, the distributed muscular loading and rhythmic characteristics of pumping may delay localized fatigue sensations through alternating recruitment patterns. Taken together, these mechanisms provide a plausible neuromuscular and perceptual framework for interpreting the observed activation–fatigue dissociation, while remaining hypotheses that require direct validation (e.g., subject-specific EMG and neurophysiological measurements).

Kinematic and ergonomic findings collectively indicate that the pumping maneuver imposes a demanding multi-planar load on the musculoskeletal system, with the lumbar region emerging as a key biomechanical constraint. In particular, the coexistence of large lumbar excursions and high stabilization demand suggests that the lumbar spine is exposed to both substantial angular displacement and high stabilization demand during pumping. This combination provides a plausible biomechanical basis for the frequent lower-back discomfort reported by sailors and may contribute to cumulative tissue stress over time, especially in the presence of the asymmetrical activation pattern observed in the simulation.

The REBA score (11; “Very High Risk”) further reinforces this interpretation by indicating an unfavorable postural and loading profile during pumping, characterized by trunk flexion/rotation, elevated shoulder postures, repetitive force production, and sustained lower-limb stabilization demands. Taken together, the kinematic, simulation, and ergonomic results support the view that pumping is associated with a substantial overuse-risk profile, particularly for the lumbar spine and shoulder–trunk kinetic chain.

Questionnaire findings were broadly consistent with this biomechanical profile, as most reported discomfort involved the lower back, shoulders, and knees, and 72.2% of participants reported pumping-related musculoskeletal discomfort. These subjective findings complement the biomechanical results and reinforce the relevance of combining model-based and self-reported measures when characterizing pumping-related load.

Although the present study focused on the pumping maneuver, our findings can be critically interpreted in light of the broader dinghy-sailing biomechanics literature, which is still predominantly centered on hiking. In line with previous reports, our results support the view that sailing-specific maneuvers impose substantial trunk and core demands, while the distribution of load across segments depends on the technical gesture being analyzed. Caraballo et al. emphasized that hiking performance is strongly influenced by anthropometric, strength, and endurance factors and noted the prolonged quasi-isometric demand on quadriceps and abdominal musculature, which is a key source of fatigue [27]. Similarly, recent training and profiling studies in hikers have shown sailing-specific lower-limb strength adaptations and improvements in trunk/lower-limb muscle activation with reduced fatigue after targeted hiking-bench training [28]. However, compared with this hiking-focused evidence, our pumping data suggest a more pronounced asymmetric contribution of the upper body/shoulder complex and trunk stabilizers, consistent with the unilateral force transmission required by the maneuver. This interpretation is also coherent with biomechanical observations showing that sailing postures are not purely static and involve multi-planar trunk motion, as well as with recent digital human modeling studies reporting measurable bilateral asymmetries and trunk/upper-limb activation patterns in Laser sailing [29].

From an applied perspective, the present findings support more targeted training and recovery strategies for sailors performing repetitive pumping maneuvers. Given the high modeled demand on deep trunk stabilizers and the observed asymmetrical loading pattern, conditioning programs should include trunk endurance training (local and global stabilizers), anti-rotation and anti-lateral-flexion exercises, and unilateral/asymmetrically loaded drills to improve force transmission capacity and tolerance to side-dominant loading. For the shoulder girdle, particularly the latissimus dorsi and scapular stabilizers, training should emphasize repeated force production with postural control (e.g., rowing/pulling patterns, scapular control, and shoulder endurance work) in order to support sail handling under fatigue. For the lower extremities, despite moderate modeled activation, the high perceived fatigue suggests the need for strength-endurance and postural stabilization training (including prolonged isometric holds and dynamic balance tasks) to improve tolerance to cumulative loading during pumping and sailing. In addition, recovery and load-management strategies (e.g., monitoring of regional fatigue/discomfort, planned recovery for lumbar/shoulder/lower-limb regions, and technique pacing/refinement) may help reduce excessive fatigue and mitigate overuse risk. These recommendations should be considered practice-oriented implications of the present biomechanical findings and warrant confirmation in intervention studies.

Several limitations should be acknowledged. First, although the land-based acquisition protocol improved measurement control and enabled accurate multi-angle postural reconstruction for the DELMIA^®^ virtual twin, it may reduce ecological validity because it does not fully reproduce on-water perturbations such as wave-induced boat motion, wind variability, and impact loading. These factors may influence joint kinematics, muscle activation strategies, and perceived fatigue during real sailing. To mitigate this limitation, the present study integrated supplementary on-water observations and post-training subjective assessments; however, the simulation outputs should still be interpreted as a standardized biomechanical reference condition rather than a complete representation of all on-water dynamics.

In addition, the musculoskeletal simulation was based on a single representative pumping cycle performed by one experienced sailor, and individual variability in technique and anthropometry was not captured. The reliance on optimization algorithms also introduces uncertainty in the estimation of muscle forces. Questionnaire data, while informative, are subject to recall bias and may not fully reflect acute physiological responses. Future research should incorporate subject-specific EMG recordings to validate and refine simulation outputs and to better characterize inter-individual variability. Longitudinal investigations examining the relationship between pumping mechanics and injury incidence, as well as intervention studies assessing the effectiveness of targeted training and ergonomic modifications, would further advance evidence-based practice in competitive sailing.

## 5. Conclusions

This study provides an integrated biomechanical and ergonomic characterization of the pumping maneuver in Laser-class sailing by combining musculoskeletal simulation, kinematic analysis, REBA, and questionnaire data from competitive sailors. Taken together, these approaches offer a multidimensional description of the objective mechanical demands and the subjective fatigue/discomfort associated with pumping.

The main findings indicate that pumping is characterized by a marked asymmetrical neuromuscular pattern, with high activation of trunk stabilizers and a clear lateral redistribution of muscular demand between the trunk and shoulder girdle. In particular, the simulation identified substantial activation of deep trunk stabilizing muscles, together with pronounced asymmetry in spinal stabilization and upper-body recruitment. Kinematic analysis further showed relevant joint excursions, with the lumbar region exhibiting the greatest motion amplitudes. The combination of high trunk muscle demand and large lumbar excursions suggests that the lumbar spine is a key biomechanical constraint during pumping and may represent a primary site of cumulative mechanical stress.

The study also highlighted a discrepancy between simulated muscle activation and perceived fatigue, as sailors reported greater fatigue in the lower limbs despite the highest simulated activation occurring in the trunk musculature. This finding suggests that subjective fatigue during pumping is influenced not only by activation magnitude, but also by sustained postural demands, balance control, and repetitive dynamic loading.

From an applied perspective, these results support the need for targeted conditioning and prevention strategies focused on trunk stabilization, asymmetrical loading tolerance, and lumbar load management. The REBA score (“Very High Risk”) further underscores the ergonomic relevance of the pumping maneuver and the need for technique refinement and risk-mitigation strategies. Although the findings are promising, they should be interpreted in light of the simulation-based design and the absence of direct EMG validation. Future studies should include experimental validation and larger on-water datasets to strengthen the transferability of these results to competitive practice.

## Figures and Tables

**Table 1:** Demographic, anthropometric, and sailing-related characteristics of the Laser sailors. Continuous variables are presented as mean ± standard deviation and observed range (minimum–maximum).

Characteristic	Value	
Number of participants	36 (28 Male; 8 Female)	
	Mean ± SD	Range
Age [year]	16.95 ± 2.63	12–25
Body mass [kg]	66.48 ± 10.50	50–96
Height [cm]	173.45 ± 7.28	160–187
Shoulder height [cm]	142.95 ± 7.08	131–157
Hip height [cm]	97.23 ± 6.53	90–113
Knee height, cm	51.68 ± 3.40	46–60
Foot size [EU]	41.48 ± 2.49	36–45
Sailing experience [year]	7.68 ± 3.77	3–15

**Table 2:** Perceived discomfort by body region during pumping and on the day after training (Borg CR-10). Values are reported as mean ± standard deviation. P-values refer to the comparison between conditions (During vs Day After) for each body region.

Body Region	During (A)	Day After (B)	*p*-Value
Neck	4.30 ± 2.34	2.97 ± 1.93	<0.001
Right Shoulder	5.36 ± 2.34	3.64 ± 1.93	<0.001
Left Shoulder	2.86 ± 1.81	2.81 ± 1.89	0.899
Right Arm	3.33 ± 2.28	3.44 ± 2.35	0.839
Left Arm	3.67 ± 2.84	3.89 ± 2.20	0.712
Right Forearm	3.86 ± 2.95	3.94 ± 2.68	0.901
Left Forearm	3.08 ± 2.86	3.33 ± 2.61	0.700
Back	4.19 ± 2.72	4.44 ± 2.50	0.686
Abdomen	4.81 ± 2.92	4.94 ± 2.33	0.824
Right Thigh	3.22 ± 2.49	3.39 ± 2.21	0.764
Left Thigh	2.89 ± 2.45	3.17 ± 2.40	0.628
Right Leg	3.36 ± 2.53	3.58 ± 2.68	0.719
Left Leg	3.36 ± 2.93	3.64 ± 2.73	0.678

**Table 3:** Joint kinematics during execution of the pumping maneuver. Values represent minimum, mean, and maximum joint angles and range of motion (ROM) across one complete pumping cycle for the 50th percentile male digital human model.

Body Region	Joint Movement	Min (°)	Mean (°)	Max (°)	ROM (°)
Upper Limb—Shoulder	Right clavicular abduction	−3.12	18.89	40.49	43.61
Upper Limb—Shoulder	Left clavicular abduction	8.73	16.34	33.56	24.83
Upper Limb—Arm	Right arm flexion	−1.81	40.84	86.37	88.18
Upper Limb—Arm	Left arm flexion	−57.47	17.39	76.17	133.64
Upper Limb—Forearm	Right forearm flexion	11.06	64.3	93.5	82.44
Upper Limb—Forearm	Left forearm flexion	42.82	107.61	139.51	96.69
Lower Limb—Hip	Right hip abduction	−14.57	8.74	27.41	41.98
Lower Limb—Hip	Left hip abduction	−11.64	5.81	45	56.64
Lower Limb—Knee	Right knee flexion	45.24	83.23	105.63	60.39
Lower Limb—Knee	Left knee flexion	20.17	73.83	105.63	85.46
Trunk—Sagittal	Trunk inclination	14.2	22.29	29.67	15.47
Trunk—Transverse	Trunk rotation	−71.7	−19.54	17.4	89.1

**Table 4:** Activation percentages of the most activated muscles during the pumping maneuver, grouped by body region.

Body Region	Muscle (DX = Right Side; SX = Left Side)	Activation (%)
Trunk	multifidus_DX	100.0
Trunk	quadratus_lumborum_DX	100.0
Trunk	obliquus_externus_DX	100.0
Trunk	Semispinalis_DX	100.0
Shoulder Left	latissimus_dorsi_SX	100.0
Trunk	psoas_major_SX	100.0
Trunk	obliquus_internus_SX	100.0
Trunk	psoas_major_DX	100.0
Trunk	erector_spinae_DX	91.8
Trunk	erector_spinae_SX	58.7
Shoulder Left	supraspinatus_SX	56.2
Upper Limb Left	triceps_SX	56.2
Upper Limb Left	biceps_SX	56.2
Shoulder Left	infraspinatus_SX	56.2
Shoulder Left	deltoideus_SX	56.2
Trunk	multifidus_SX	48.9
Lower Limb Right	tensor_fasciae_latae_DX	45.5
Lower Limb Right	poplitues_DX	45.5
Lower Limb Right	plantaris_DX	45.5
Lower Limb Right	gracilis_DX	45.5
Lower Limb Right	semimembranosus_DX	45.5
Lower Limb Right	sartorius_DX	45.5
Lower Limb Right	biceps_femoris_DX	45.5
Lower Limb Right	peroneus_tertius_DX	45.5
Lower Limb Right	tibialis_anterior_DX	45.5
Shoulder Left	serratus_anterior_SX	43.2
Shoulder Left	trapezius_SX	43.2
Trunk	rectus_abdominis	34.1
Neck	sternoclaidomastoid_SX	33.1
Trunk	transversus	32.5
Lower Limb Right	adductor_brevis_DX	26.6
Lower Limb Right	pectineus_DX	26.6
Lower Limb Right	obturator_internus_DX	26.6
Lower Limb Right	obturator_externus_DX	26.6
Lower Limb Right	gemellus_superior_DX	26.6
Lower Limb Right	gemellus_inferior_DX	26.6
Lower Limb Right	quadratus_femoris_DX	26.6
Lower Limb Right	adductor_longus_DX	26.6
Lower Limb Right	piriformis_DX	26.6
Lower Limb Right	gluteus_medius_DX	26.6
Lower Limb Right	gluteus_minimus_DX	26.6
Lower Limb Right	iliacus_DX	26.6
Lower Limb Right	gastrocnemius_DX	26.6

## Data Availability

The data presented in this study are available on request from the corresponding author.
